# Burnout among the podiatry profession: A survey of podiatrists in Aotearoa New Zealand

**DOI:** 10.1002/jfa2.12030

**Published:** 2024-06-10

**Authors:** Mia Clarke, Mike Frecklington, Sarah Stewart

**Affiliations:** ^1^ Faculty of Health and Environmental Sciences School of Clinical Sciences Auckland University of Technology Auckland New Zealand; ^2^ Active Living and Rehabilitation: Aotearoa New Zealand Health and Rehabilitation Research Institute School of Clinical Sciences Auckland University of Technology Auckland New Zealand

**Keywords:** burnout, Maslach burnout inventory, occupational stress, podiatry, professional wellbeing

## Abstract

**Introduction:**

Burnout and occupational stress have not yet been explored within the Aotearoa New Zealand (AoNZ) podiatry workforce despite research suggesting an increased risk among this population. This study aimed to: (i) determine the prevalence and severity of burnout risk and occupational stress among AoNZ podiatrists; (ii) determine the factors associated with burnout risk and occupational stress among AoNZ podiatrists; and (iii) examine the relationship between burnout risk and occupational stress.

**Methods:**

A cross‐sectional online survey study was undertaken involving registered podiatrists practicing in AoNZ. Personal and professional demographic characteristics were captured. Participants also completed the Maslach Burnout Inventory (assessing three domains of emotional exhaustion, depersonalisation and personal accomplishment) and the Workplace Stress Scale as measures of burnout risk and occupational stress, respectively. Descriptive statistics, multiple regression analyses and correlation analyses were performed to address the research aims.

**Results:**

Responses from 112 AoNZ podiatrists were included in the analyses. High levels of emotional exhaustion were identified in 43.8% of practitioners and were associated with physical activity status, sector of work, working in isolation and work hours (*R* ^2^ = 0.304, *F* (8, *N* = 110) = 5.519, *p* < 0.001). High levels of depersonalisation were seen in 13.4% of practitioners and were associated with patient caseload and work hours, (*R* ^2^ = 0.183, *F* (4, *N* = 108) = 5.770, *p* < 0.001). Low levels of personal accomplishment were observed in 8.9% of practitioners and associated with ethnicity, physical activity status and patient caseload, (*R* ^2^ = 0.152, *F* (5, *N* = 106) = 3.577, *p* < 0.005). A total of 27.7% of practitioners exhibited an overall moderate to high risk of developing burnout. Over a fifth of practitioners exhibited stress at severe or dangerous levels. Stress levels were significantly associated with physical activity status, sector of work and management responsibility, (*R* ^2^ = 0.282, *F* (5, *N* = 47) = 3.218, *p* = 0.15). A strong positive relationship was found between emotional exhaustion and stress (*rho* = 0.59, *p* < 0.001).

**Conclusions:**

The findings reflect a moderate to severe risk of developing burnout within the workforce, with high workloads and collegial isolation constituting the primary modifiable factors driving burnout development. To maintain retention and well‐being within the workforce, mitigation strategies must be implemented to address this issue.

## 1 INTRODUCTION

Burnout within the medical context is a frequently discussed topic that, over recent decades, has become an increased threat to healthcare workers and systems [1]. Burnout is a behavioural and emotional impairment in response to high‐level exposure to occupational‐related stress [2]. Burnout is typically characterised by three distinct domains including emotional exhaustion (the state of being emotionally drained and the depletion of emotional resources), depersonalisation/cynicism (negative and detached responses to other people, including colleagues and patients) and reduced personal accomplishment (a decline in feelings of competence and performance in the ability to perform one's job) [3]. While burnout development stems from long‐term emotional and interpersonal workplace stressors, occupational stress should be considered a separate concept, yet an underlying driver to burnout development [4]. Occupational stress can be described as the harmful physical and emotional responses that occur when the job requirements do not match the worker's capabilities, resources or needs [5]. Transient or mild instances of work‐related stress are expected and generally managed. However, sustained or elevated levels of work‐related stress without effective coping strategies may elicit burnout development [6, 7].

The Aotearoa New Zealand (AoNZ) podiatry workforce is a small yet essential element of allied healthcare delivery, with a reported 474 podiatrists actively practicing in 2023 [8]. However, previous studies investigating AoNZ, Australian and UK podiatrists have indicated that burnout is a real and concerning issue within the current climate [9, 10, 11]. Significant retention and recruitment difficulties have been unveiled, with findings that AoNZ would need to more than double its current workforce numbers to match the per‐capita ratio of practicing podiatrists in Australia and the UK [9]. Mandy and Tinley (2004) conducted a study comparing levels of burnout in newly qualified practitioners in Australia and the UK, finding that levels of burnout across these populations were higher than previously indicated [10]. More recently, Couch et al. (2023) performed a cross‐sectional online survey study among Australian podiatrists, concluding over a third of respondents were at risk of burnout [11]. The evident shortage of AoNZ podiatrists impels increases in workload and stressors, placing AoNZ podiatrists at a heightened risk of burnout compared to their already burnt out international counterparts.

Burnout risk among AoNZ's podiatrists may be particularly prevalent among rural practitioners due to podiatry workforce shortages outside of major cities [12]. Workforce shortages and burnout among rural practitioners is likely exacerbated by a number of factors including professional isolation, limited support networks, limited resources and high workloads [13].

The predicted rise of podiatry care demands poses additional risk to the sustainability and well‐being of the workforce. By 2034, it is predicted 20% of AoNZ's population will be aged 65+ [14]. AoNZ's Ministry of Health (MOH) acknowledges that an increase in the older population means an increase in health care needs, chronic health conditions and disabilities requiring ongoing and regular support [15]. Parallel to an ageing population, the prevalence of diabetes is expected to rise significantly. An estimated 228,000 people in AoNZ live with Type 2 diabetes mellitus alone, with a predicted growth to approximately 400,000 by 2040 [16]. The AoNZ MOH reports an increase in the prevalence of diabetes within deprived areas where fewer foot care services are available [17]. Moreover, diabetic rates among Indigenous Māori and Pasifika populations are disproportionately higher with poorer health outcomes, consequently magnifying the inequities within AoNZ's healthcare system [16].

**FIGURE 1:**
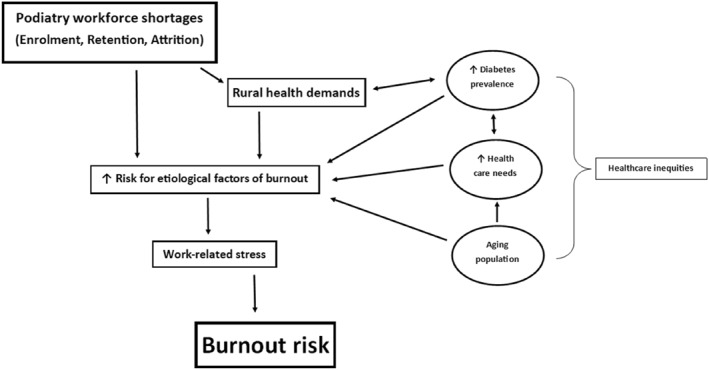
Summary of the current challenges faced by the AoNZ podiatry profession. Original figure. AoNZ, Aotearoa New Zealand.

An understaffed workforce supporting these demands jeopardises the delivery of gold‐standard care to all New Zealanders while placing podiatrists at a heightened risk for the development of burnout [18]. A simplified summary of these current challenges faced by the profession and their direct link to burnout risk is outlined in Figure 1. Although the current climate and challenges AoNZ's podiatrists face reflects a profession under immense pressure, it is unknown how many practitioners experience occupational stress or are at risk of developing burnout. A comprehensive, quantitative investigation into burnout and stress among AoNZ podiatrists is imperative to inform individual organisational and system‐level policy, interventions and investments [9, 19]. This study will address the following aims:

**Aim 1:** To determine the prevalence and severity of burnout risk and occupational stress among podiatrists in AoNZ
**Aim 2:** To determine the factors associated with burnout risk and occupational stress among podiatrists in AoNZ
**Aim 3:** To examine the relationship between burnout risk and occupational stress among podiatrists in AoNZ

## 2 MATERIALS AND METHODS

### 2.1 Design, setting and population

This was a cross‐sectional study of podiatrists practicing in AoNZ. Data were collected from March 2023 to May 2023 via a single self‐administered questionnaire through the Qualtrics online survey platform. Practitioners were invited to participate in the study through advertisements in the Podiatry NZ monthly newsletter and the NZ Podiatry Alumni Facebook page. At the time of data collection, there were 390 Podiatry NZ members and 550 members on the Facebook group [20]. Only practitioners who were registered with the Podiatrists Board of NZ, held a current Annual Practicing Certificate and were currently working within AoNZ were invited to participate. Eligibility of participants was self‐declared. This study was approved by the Auckland University of Technology Ethics Committee (AUTEC), reference 22/368.

### 2.2 Data collection

The survey comprised four sections: participant information page and the informed consent process, participants' personal demographic information, participants' professional demographic details and burnout and occupational stress measurement questionnaires. The complete survey is provided in Supporting Information S1.

Personal and professional demographic factors were selected based on factors identified as possible contributors to burnout among other allied health professionals [21, 22, 23]. The Maslach Burnout Inventory Human Services Survey (MBI‐HSS) assessed risk for burnout development. The inventory contains 22 items across three domains: emotional exhaustion (nine items), depersonalisation (five items) and personal accomplishment (eight items) [24]. The items are ranked on a seven‐point Likert scale with scores summated to provide total scores for each domain to provide a measure of burnout risk severity. Continuous and categorical measures were used to measure the prevalence of low, moderate and high burnout risk. Scores were calculated using the following cut‐off values outlined in the Maslach Burnout Inventory (MBI) Manual (1997): <16 (low), 17–26 (moderate) and >27 (high) for the emotional exhaustion domain; <6 (low), 7–12 (moderate) and >13 (high) for the depersonalisation domain and >38 (low), 33–37 (moderate), <32 (high) for the personal accomplishment domain. High scores for emotional exhaustion and depersonalisation and low scores for personal accomplishment indicate high overall risk for developing burnout [24]. Moderate scores for emotional exhaustion, depersonalisation and personal accomplishment indicate moderate overall risk for developing burnout. Low scores for emotional exhaustion and depersonalisation and high scores for personal accomplishment indicate low overall risk for developing burnout. Importantly, the MBI was not developed as a diagnostic tool for burnout, but rather to assess the risk of burnout development [24]. Therefore, for the purpose of the current study these categorical scores were considered indicative of low, moderate and high risk of developing burnout, respectively.

The Workplace Stress Scale (WSS) developed by the American Institute of Stress was used to assess occupational stress levels [25]. The instrument includes 10 items with each item rated on a 5‐point Likert scale producing both continuous and categorical scores. Total scores are summated to classify overall stress levels as relatively calm (≤15), fairly low (16–20), moderate (21–25), severe (26–30) and potentially dangerous (31–40) [25].

### 2.3 Analysis

To address Aim 1, descriptive statistics (*n*, %) were reported to show the proportion of participants who had a low, moderate and high risk for developing burnout in each of the three burnout domains and the prevalence of high overall burnout risk. The mean (SD) for continuous measures of burnout in each of the three MBI domains was also reported as a measure of burnout severity. Similarly, *n* (%) was reported to determine the prevalence of various levels of occupational stress among participants (as determined by the WSS), with the mean (SD) scores also used to indicate severity of overall stress.

To address Aim 2, bivariable analyses were performed between the continuous measures of burnout for each of the three domains, overall stress and all independent variables to establish empirical relationships. The continuous measures of burnout were used in this analysis due to the lack of diagnostic validity in using cut‐points. Descriptive statistics were reported for all variables, including mean (SD) and median (IQR) values. A parametric analysis of variance (or the non‐parametric Kruskal–Wallis *H* test) was used to determine the association between continuous measures of burnout risk and stress and the categorical independent variables. Additionally, a two‐tailed independent samples *t*‐test (or the non‐parametric Mann–Whitney *U* test) was conducted to determine the association between the continuous measure of burnout risk and stress and the dichotomous independent variables. Following confirmation of no violation in normality, homogeneity or collinearity of variables, four multiple regression analyses were performed to determine which combination of independent variables contributed most to the prediction of each burnout domain and stress. Demographic and professional characteristics in the bivariable analysis achieving a *p* value < 0.10 were included in the multiple regression analysis [26]. Variables with insufficient data were excluded to eliminate violating normality assumptions.

To address Aim 3, the parametric Pearson's *r* (or non‐parametric Spearman's *rho*) was conducted to determine the associations between burnout risk and stress. Correlation coefficients were reported as *r* or *rho* with indications of respective significance levels. Interpretation of variable strength was informed by Cohen (1988) using the following guidelines: small *r* = 0.10–0.29; medium *r* = 0.30–0.49; large *r* = 0.50–1.00 [27].

All inferential analyses were conducted in IBM SPSS Statistics v.25 with *p* values < 0.05 considered statistically significant, unless otherwise stated. Due to the exploratory nature of this study, no adjustments were made for multiplicity, however all test statistics, estimates and their significance levels were reported. Pairwise deletion was used to handle missing values.

## 3 RESULTS

### 3.1 Participant characteristics

A total of 131 podiatrists responded to the survey, however, due to incomplete data sets, data from only 112 participants were included for analysis. The minimum information required for the inclusion of data sets consisted of demographic information and the complete MBI‐HSS. This represents a completion rate of 85.5% and approximately 23.6% of the AoNZ 2022 podiatry workforce [8]. Table 1 displays a summary of participant personal and professional demographic characteristics. The majority of respondents were aged between 31 and 40 years, identified as NZ European, confirmed no religious affiliation and had between two to four dependants. Most respondents slept between six to 8 hours per night, participated in regular physical activity and did not regularly practice mindfulness. The majority of participants had been practicing for more than 20 years, worked in private practice, owned their workplace, worked between 40 and 50 h per week and had a daily commute time of less than 10 minutes. Over a third of respondents worked alone as sole practitioners, with the highest volume of participation from the rural North Island of AoNZ.

**TABLE 1:** Participant personal and professional demographic characteristics (*n* = 112).

Variable	Summary statistics, *n* (%)
Gender	
Male	25 (22.3%)
Female	85 (75.9%)
Prefer to self‐describe	2 (1.8%)
Age	
20–30 years	24 (21.4%)
31–40 years	36 (32.1%)
41–50 years	30 (26.8%)
>50 years	22 (19.6%)
Ethnicity	
NZ European	78 (69.6%)
Māori	6 (5.4%)
Asian	12 (10.7%)
Other	16 (14.3%)
Marital status	
Single (never married)	19 (17.0%)
Married/de facto	87 (77.7%)
Divorced/separated	4 (3.6)
Prefer not to say	2 (1.8)
Number of dependants	
0	48 (42.9%)
1	8 (7.1%)
2–4	50 (44.6%)
>4	5 (4.5%)
Prefer not to say	1 (0.9%)
Sleep/night	
<6 h	15 (13.4%)
6–8 h	83 (74.1%)
>8 h	14 (12.5%)
Religion affiliation	
None	75 (67.0%)
Religious	37 (33.0%)
Christian	16 (14.3%)
Catholic	7 (6.3%)
Other	9 (8.0%)
Physical activity/week	92 (82.1%)
<2 h	19 (17%)
3–4 h	35 (31.3%)
>4 h	38 (33.9%)
Mindfulness practice	
No	73 (65.2%)
Yes	39 (34.8%)
Mindfulness practice/week	
0.25–1 h	16 (14.3%)
1–2 h	17 (15.2%)
>2 h	6 (5.4%)
Practice experience	
0–5 years	17 (15.2%)
5–10 years	28 (25%)
10–15 years	16 (14.3%)
15–20 years	11 (9.8%)
>20 years	40 (35.7%)
Education	
Bachelor's degree	61 (54.5%)
Postgraduate	40 (35.7%)
Master's degree	5 (4.5%)
Doctorate	6 (5.4%)
Current postgraduate student	
Yes	14 (12.5%)
No	98 (87.5%)
Sector	
Private practice	87 (77.7%)
Public	7 (6.3%)
Research/Education	5 (4.5%)
Combination	13 (11.6%)
Workplace owner	
No	48 (42.9%)
Yes	64 (57.1%)
Additional management responsibilities	
Yes	19 (17.0%)
No	93 (83.0%)
Collegial isolation (works with)	
Alone	43 (38.4%)
Works with others	69 (61.6%)
Work environment	
Clinic	96 (85.7%)
Out of clinic	11 (9.8%)
Nonclinical	5 (4.5%)
Patient caseload^a^	
Multi‐speciality	46 (41.1%)
One or two specialities	62 (55.4%)
No patient contact	4 (3.6%)
Work hours (per week)	
<30 h	13 (11.6%)
30–40 h	42 (37.5%)
40–50 h	44 (39.3%)
>50 h	13 (11.6%)
Daily patient contact	
<4 h	8 (7.1%)
4–7 h	62 (55.4%)
>7 h	42 (37.5%)
Region of practice	
Auckland	29 (25.9%)
Wellington	13 (11.6%)
Christchurch	25 (22.3%)
North Island rural	33 (29.5%)
South Island rural	12 (10.7%)
Daily commute time (mins)	
<10 min	37 (33.0%)
10–20 min	18 (16.1%)
20–30 min	19 (17.0%)
30–60 min	18 (16.1%)
>60 min	20 (17.9%)
Gross annual income	
<$60k	18 (16.1%)
$60K–$80k	38 (33.9%)
$80k–$100k	28 (25.0%)
>$100k	28 (25.0%)
Gross household annual income	
<$100k	27 (24.1%)
$100k–$150k	34 (30.4%)
$150k–$200k	28 (25.0%)
$200k–$250k	13 (11.6%)
>$250k	10 (8.9%)

^a^ Specialities = dermatology or musculoskeletal or high‐risk (rheumatology/diabetes).

### 3.2 Aim 1: Prevalence and severity of burnout risk and occupational stress

Table 2 displays the prevalence and severity of burnout risk and occupational stress according to the MBI‐HSS and WSS respectively. Based on the categorisation of the MBI scores, the majority of respondents demonstrated high levels of emotional exhaustion (43.8%), while 13.4% had high levels of depersonalisation and 8.9% had low levels of personal accomplishment. Overall risk of burnout development was high among 4.5% of respondents; moderate among 23.3% and low among 72.1%. According to the WSS, one third of respondents demonstrated moderate stress levels, with over a fifth of respondents exhibiting stress at severe or dangerous levels.

**TABLE 2:** Descriptive statistics for prevalence and severity of burnout risk and occupational stress (*n* = 112).

Variable	Summary statistics
MBI—Emotional Exhaustion	
Mean (SD)	25.32 (11.96)
Low, *n* (%)	26 (23.2%)
Moderate, *n* (%)	37 (33.0%)
High, *n* (%)	49 (43.8%)
MBI—Depersonalisation	
Mean (SD)	6.08 (5.88)
Low, *n* (%)	74 (66.1%)
Moderate, *n* (%)	23 (20.5%)
High, *n* (%)	15 (13.4%)
MBI—Personal accomplishment^a^	
Mean (SD)	39.41 (5.82)
Low, *n* (%)	10 (8.9%)
Moderate, *n* (%)	22 (19.6%)
High, *n* (%)	80 (71.4%)
MBI—overall burnout risk^b^	
High, *n* (%)	5 (4.5%)
Moderate, *n* (%)	26 (23.2%)
Low, *n* (%)	81 (72.3%)
Workplace stress scale (WSS)^c^	
Mean (SD)	21.03 (6.87)
Calm, *n* (%)	23 (20.5%)
Fairly low, *n* (%)	28 (25.0%)
Moderate, *n* (%)	37 (33.0%)
Severe, *n* (%)	11 (9.8%)
Dangerous, *n* (%)	12 (10.7%)

Abbreviation: MBI = Maslach burnout inventory.

^a^ Low personal accomplishment is considered to be associated with increased burnout risk.

^b^ High scores for emotional exhaustion and depersonalisation and low scores for personal accomplishment indicate a high overall risk for the development of burnout. Moderate scores for emotional exhaustion, depersonalisation, and personal accomplishment indicate moderate overall risk for developing burnout. Low scores for emotional exhaustion and depersonalisation, and high scores for personal accomplishment indicate low overall risk for developing burnout.

^c^ Data was available for 111 participants.

### 3.3 Aim 2: Factors associated with burnout risk and occupational stress

Complete results from the bivariable and multiple regression analyses can be found in Table 3 and Table 4 respectively.

**TABLE 3:** Bivariable analyses of factors associated with burnout domains and total Workplace Stress Scale (WSS) score.

	Variable	Emotional exhaustion			Depersonalisation			Personal accomplishment			WSS		
		Mean (SD)	Median (IQR)	*p*	Mean (SD)	Median (IQR)	*p*	Mean (SD)	Median (IQR)	*p*	Mean (SD)	Median (IQR)	*p*
Sex	Male	22.60 (13.33)	21.00 (18.00)	0.21^a^	4.88 (4.05)	4.00 (6.00)	0.61^b^	39.36 (3.96)	40.00 (7.00)	0.39^b^	20.63 (5.73)	20.50 (6.00)	0.75^a^
	Female	26.05 (11.62)	25.00 (17.00)		6.16 (5.94)	5.00 (7.00)		39.55 (6.28)	40.00 (7.00)		21.14 (7.18)	21.00 (9.00)	
Age (years)	20–30	25.42 (10.67)	26.50 (17.00)	0.97^a^	7.00 (5.45)	5.50 (11.00)	0.52^b^	39.33 (5.24)	41.00 (7.00)	0.19^b^	20.58 (7.06)	20.50 (7.00)	0.93^a^
	31–40	25.75 (13.48)	24.50 (18.00)		6.42 (6.74)	5.00 (5.00)		38.06 (6.08)	38.00 (7.00)		21.06 (7.83)	19.00 (9.00)	
	41–50	24.43 (11.52)	21.00 (17.00)		4.40 (3.49)	4.00 (5.00)		41.13 (3.85)	41.00 (5.00)		21.67 (5.13)	21.00 (5.00)	
	>50	25.73 (11.96)	26.50 (19.00)		6.82 (7.21)	3.50 (10.00)		39.36 (7.69)	39.50 (10.00)		20.59 (7.47)	23.00 (11.00)	
Ethnicity	NZ European	25.65 (11.96)	23.50 (15.00)	0.51^b^	5.99 (6.01)	4.00 (7.00)	0.39^b^	40.56 (4.99)	41.00 (6.00)	0.021^b^	20.83 (6.75)	21.00 (8.00)	0.17^b^
	Māori	21.50 (14.83)	16.00 (30.00)		4.33 (5.05)	1.50 (8.00)		34.83 (9.83)	38.00 (20.00)		21.50 (7.40)	20.50 (11.00)	
	Asian	22.25 (10.19)	23.00 (20.00)		6.17 (5.57)	3.50 (9.00)		36.50 (4.66)	36.00 (8.00)		18.00 (5.62)	18.00 (9.00)	
	Other	27.44 (12.44)	27.50 (18.00)		7.13 (6.04)	5.50 (4.00)		37.69 (6.99)	38.50 (9.00)		23.88 (7.54)	23.00 (13.00)	
Marital status	Single	27.26 (10.79)	29.00 (15.00)	0.38^a^	6.39 (5.57)	6.00 (8.00)	0.67^b^	39.57 (4.79)	40.00 (8.00)	0.78^b^	22.96 (8.82)	22.00 (14.00)	0.15^a^
	Married/de facto	24.77 (12.38)	22.00 (18.00)		5.99 (6.05)	4.00 (6.00)		39.46 (6.10)	40.00 (7.00)		20.63 (6.25)	21.00 (7.00)	
Number of dependants	0	24.81 (11.42)	24.50 (17.00)	0.97^b^	6.85 (6.46)	5.00 (9.00)	0.81^b^	39.35 (5.73)	40.50 (8.00)	0.69^b^	20.36 (7.47)	19.00 (10.00)	0.39^b^
	1	23.38 (8.75)	27.00 (16.00)		4.63 (3.42)	4.50 (6.00)		36.50 (7.78)	39.00 (13.00)		21.50 (4.87)	22.00 (6.00)	
	2–4	25.86 (13.07)	22.00 (21.00)		5.56 (5.73)	3.50 (6.00)		40.12 (5.17)	40.50 (7.00)		21.92 (6.84)	21.00 (8.00)	
	>4	27.60 (13.61)	18.00 (25.00)		5.80 (5.45)	4.00 (11.00)		38.80 (9.20)	42.00 (14.00)		19.00 (2.74)	19.00 (5.00)	
Sleep/night (hours)	<6	23.80 (15.20)	18.00 (24.00)	0.17^b^	6.73 (6.93)	5.00 (5.00)	0.21^b^	37.67 (6.87)	41.00 (9.00)	0.47^b^	21.57 (8.27)	20.50 (10.00)	0.16^b^
	6–8	26.31 (11.20)	26.00 (17.00)		6.20 (5.58)	5.00 (7.00)		39.64 (5.39)	40.00 (7.00)		21.40 (6.67)	21.00 (8.00)	
	>8	21.07 (12.35)	20.50 (18.00)		4.64 (6.63)	3.50 (5.00)		39.93 (7.12)	42.00 (9.00)		18.29 (6.39)	17.00 (8.00)	
Religious	Yes	27.78 (12.76)	29.00 (20.00)	0.11^a^	6.78 (6.62)	5.00 (8.00)	0.54^b^	38.84 (6.61)	40.00 (9.00)	0.63^b^	22.22 (7.16)	22.00 (9.00)	0.24^a^
	No	23.87 (11.51)	21.00 (17.00)		5.75 (5.64)	4.00 (5.00)		39.70 (5.44)	40.00 (6.00)		20.56 (6.73)	21.00 (7.00)	
Physically active	Yes	23.70 (11.51)	21.50 (17.00)	0.006^a^	5.98 (5.84)	4.00 (7.00)	0.94^b^	39.96 (5.69)	41.00 (6.00)	0.015^b^	20.29 (6.83)	20.00 (8.00)	0.037^a^
	No	31.94 (11.11)	35.00 (13.00)		5.50 (4.64)	4.50 (5.00)		36.61 (6.08)	36.50 (7.00)		24.00 (5.98)	22.00 (7.00)	
Physical activity/week (hours)	<2	25.58 (12.17)	25.00 (22.00)	0.74^b^	4.84 (3.93)	4.00 (5.00)	0.89^b^	38.74 (7.05)	41.00 (8.00)	0.43^b^	23.00 (7.62)	23.00 (11.00)	0.20^b^
	3–4	23.11 (10.95)	21.00 (18.00)		6.77 (6.69)	5.00 (10.00)		39.46 (5.69)	40.00 (5.00)		19.63 (5.93)	19.00 (6.00)	
	>4	23.29 (11.89)	21.00 (15.00)		5.82 (5.83)	4.00 (6.00)		41.03 (4.84)	41.50 (8.00)		19.51 (7.04)	21.00 (11.00)	
Mindfulness practice	Yes	25.79 (10.73)	25.00 (14.00)	0.51^b^	6.10 (4.70)	5.00 (6.00)	0.34^b^	39.74 (6.03)	40.00 (8.00)	0.59^a^	20.74 (6.94)	19.00 (9.00)	0.71^a^
	No	25.07 (12.62)	23.00 (21.00)		6.07 (6.45)	4.00 (6.00)		39.23 (5.73)	40.00 (8.00)		21.18 (6.87)	21.00 (7.00)	
Mindfulness practice/week (hours)	0.25–1	24.38 (11.23)	24.50 (14.00)	0.21^b^	6.25 (3.92)	5.50 (5.00)	0.67^b^	40.31 (6.74)	41.50 (7.00)	0.020^b^	20.44 (8.01)	18.50 (9.00)	0.16^b^
	1–2	28.88 (9.83)	30.00 (17.00)		6.18 (4.98)	5.00 (9.00)		37.65 (5.28)	37.00 (7.00)		22.47 (5.74)	23.00 (9.00)	
	>2	20.83 (10.83)	21.00 (15.00)		5.50 (6.44)	3.50 (8.00)		44.17 (3.43)	45.50 (6.00)		16.67 (6.15)	15.00 (12.00)	
Practice experience (years)	0–5	26.35 (9.85)	29.00 (14.00)	0.82^b^	6.71 (6.27)	3.00 (13.00)	0.78^b^	39.29 (4.99)	40.00 (8.00)	0.15^b^	21.94 (7.11)	22.00 (10.00)	0.87^b^
	5–10	23.82 (11.72)	21.00 (20.00)		5.86 (5.08)	5.00 (6.00)		37.07 (7.13)	39.00 (9.00)		20.19 (7.10)	19.00 (7.00)	
	10–15	24.94 (14.43)	24.50 (17.00)		7.75 (8.32)	4.50 (7.00)		40.75 (4.97)	41.00 (7.00)		20.88 (8.44)	20.00 (11.00)	
	15–20	24.73 (14.69)	21.00 (31.00)		3.55 (2.30)	4.00 (4.00)		38.73 (3.82)	38.00 (6.00)		21.36 (6.45)	21.00 (7.00)	
	>20	26.25 (11.55)	24.50 (18.00)		6.00 (5.76)	4.00 (7.00)		40.75 (5.56)	41.00 (7.00)		21.18 (6.30)	21.00 (8.00)	
Education	Bachelors	25.98 (12.03)	27.00 (18.00)	0.28^b^	6.79 (6.07)	5.00 (9.00)	0.15^b^	39.28 (5.51)	40.00 (7.00)	0.23^b^	20.10 (7.06)	19.50 (9.00)	0.21^b^
	Post‐graduate	25.65 (11.01)	25.00 (16.00)		5.73 (6.01)	4.00 (5.00)		38.93 (6.62)	40.00 (9.00)		21.63 (5.80)	21.00 (6.00)	
	Masters/doctorate	20.45 (14.69)	17.00 (12.00)		3.45 (3.21)	3.00 (5.00)		41.91 (3.78)	42.00 (6.00)		23.91 (8.80)	25.00 (17.00)	
Current postgraduate student	Yes	24.14 (11.85)	26.50 (14.00)	0.70^a^	5.36 (4.52)	3.50 (6.00)	0.83^b^	38.86 (5.41)	40.50 (6.00)	0.65^b^	19.93 (4.05)	21.00 (4.00)	0.79^b^
	No	25.49 (12.02)	24.00 (18.00)		6.18 (6.06)	4.00 (7.00)		39.49 (5.89)	40.00 (8.00)		21.19 (7.18)	21.00 (9.00)	
Sector	Private practice	25.16 (11.42)	25.00 (15.00)	0.08^b^	6.38 (6.26)	4.00 (7.00)	0.38^b^	39.38 (6.03)	40.00 (8.00)	0.78^b^	19.79 (6.30)	19.50 (7.00)	0.007^b^
	Public	24.57 (13.55)	22.00 (28.00)		5.00 (3.92)	4.00 (5.00)		37.29 (7.87)	41.00 (9.00)		26.00 (8.72)	28.00 (14.00)	
	Research/education	13.60 (9.81)	16.00 (19.00)		2.60 (3.21)	1.00 (5.00)		41.80 (3.42)	41.00 (6.00)		21.80 (7.30)	21.00 (12.00)	
	Combin‐ation	31.31 (12.94)	29.00 (22.00)		6.00 (4.66)	5.00 (7.00)		39.85 (3.44)	41.00 (6.00)		26.23 (6.38)	26.00 (10.00)	
Workplace owner	Yes	26.62 (12.20)	26.00 (18.00)	0.18^a^	6.75 (6.57)	5.00 (6.00)	0.21^b^	39.56 (6.24)	40.00 (9.00)	0.60^b^	20.42 (6.35)	20.00 (8.00)	0.14^a^
	No	23.58 (11.52)	22.50 (16.00)		5.19 (4.73)	3.00 (6.00)		39.21 (5.26)	40.00 (7.00)		21.85 (7.51)	21.00 (9.00)	
Additional management responsibility	Yes	26.11 (11.84)	24.00 (22.00)	0.35^b^	6.00 (4.63)	5.00 (7.00)	0.15^b^	39.37 (3.76)	40.00 (4.00)	0.70^b^	25.22 (7.27)	23.50 (10.00)	0.039^b^
	No	21.93 (11.21)	22.00 (17.00)		4.66 (4.79)	2.00 (6.00)		39.10 (6.11)	41.00 (8.00)		19.76 (6.97)	21.00 (9.00)	
Collegial isolation	Alone	29.84 (13.05)	30.00 (24.00)	0.003^a^	7.00 (5.94)	5.00 (7.00)	0.063^b^	37.79 (6.53)	38.00 (9.00)	0.016^b^	21.98 (6.82)	21.00 (6.00)	0.29^b^
	With other health practitioners	22.51 (10.35)	21.00 (15.00)		5.51 (5.81)	3.00 (6.00)		40.42 (5.11)	41.00 (6.00)		20.43 (6.88)	20.50 (9.00)	
Work environment	Clinic	25.86 (11.89)	25.00 (18.00)	0.11^b^	6.48 (6.12)	4.50 (7.00)	0.13^b^	39.27 (6.03)	40.00 (8.00)	0.69^b^	21.21 (7.06)	21.00 (7.62)	0.61^a^
	Out of clinic	25.91 (11.54)	24.00 (22.00)		4.18 (3.49)	3.00 (7.00)		39.55 (4.72)	41.00 (7.00)		19.09 (4.89)	19.00 (5.00)	
	Non‐clinical	13.60 (9.81)	16.00 (19.00)		2.60 (3.21)	1.00 (5.00)		41.80 (3.42)	41.00 (6.00)		21.80 (7.29)	21.00 (12.00)	
Patient caseload	Multi‐speciality	27.22 (13.46)	28.50 (21.00)	0.38^b^	8.26 (6.73)	7.00 (8.00)	<0.001^b^	38.07 (6.55)	39.00 (8.00)	0.06^b^	21.80 (7.13)	21.00 (9.00)	0.30^a^
	One or two specialities	24.84 (10.29)	23.00 (14.00)		4.69 (4.74)	3.00 (4.00)		40.23 (5.16)	41.00 (6.00)		20.40 (6.63)	20.50 (7.00)	
Work hours/week	<30 h	16.85 (8.01)	17.00 (9.00)	0.004^b^	2.38 (2.02)	2.00 (4.00)	0.049^b^	39.15 (8.06)	42.00 (12.00)	0.87^b^	19.15 (5.70)	21.00 (8.00)	0.12^b^
	30–40 h	23.26 (11.14)	23.50 (18.00)		6.36 (5.56)	4.50 (8.00)		39.69 (5.50)	41.00 (7.00)		19.37 (6.15)	21.00 (9.00)	
	40–50 h	27.57 (11.32)	28.00 (20.00)		5.91 (4.58)	4.50 (7.00)		39.39 (5.50)	40.00 (7.00)		21.86 (6.74)	21.00 (7.00)	
	>50 h	32.85 (14.21)	30.00 (27.00)		9.46 (10.37)	6.00 (17.00)		38.85 (5.97)	39.00 (11.00)		25.31 (8.66)	27.00 (16.00)	
Daily patient contact	<4 h	20.38 (14.69)	18.00 (27.00)	0.18^b^	4.38 (6.02)	2.00 (6.00)	0.11^b^	39.63 (3.93)	38.50 (6.00)	0.84^b^	22.75 (5.83)	24.00 (6.00)	0.28^b^
	4–7 h	24.29 (11.03)	22.00 (16.00)		5.58 (5.52)	3.50 (5.00)		39.65 (5.72)	40.50 (7.00)		20.31 (7.08)	21.00 (9.00)	
	>7 h	27.79 (12.51)	27.50 (19.00)		7.14 (6.33)	5.00 (7.00)		39.02 (6.33)	40.00 (10.00)		21.74 (6.75)	21.00 (8.00)	
Region of practice	Urban	24.01 (11.49)	23.00 (16.00)	0.18^b^	6.09 (5.81)	5.00 (6.00)	0.97^b^	40.01 (4.90)	40.00 (5.00)	0.46^b^	21.21 (6.82)	21.00 (8.00)	0.73^a^
	Rural	27.27 (12.49)	28.00 (20.00)		6.07 (6.05)	4.00 (7.00)		38.51 (6.92)	40.00 (10.00)		20.75 (7.01)	21.00 (8.00)	
Daily commute	<10 min	26.16 (12.78)	22.00 (17.00)	0.11^b^	6.46 (6.52)	4.00 (8.00)	0.66^b^	39.51 (7.09)	41.00 (8.00)	0.32^b^	20.54 (6.70)	19.00 (8.00)	0.55^b^
	10–30 min	21.83 (9.65)	21.00 (16.00)		4.97 (4.43)	3.00 (6.00)		40.33 (4.93)	40.00 (6.00)		20.09 (6.38)	21.00 (9.00)	
	30–60 min	24.61 (12.34)	27.50 (18.00)		7.78 (7.81)	6.00 (7.00)		39.56 (4.49)	39.50 (7.00)		21.22 (8.20)	22.00 (14.00)	
	>60 min	30.05 (12.67)	32.00 (23.00)		5.75 (4.99)	5.00 (6.00)		37.70 (5.75)	38.50 (7.00)		22.70 (6.28)	21.00 (7.00)	
Gross annual income	<$60k	27.83 (13.24)	26.50 (25.00)	0.52^b^	7.83 (6.68)	7.50 (10.00)	0.21^b^	36.78 (6.89)	38.50 (8.00)	0.28^b^	22. 06 (8.31)	21.50 (13.00)	0.81^a^
	$60k–$80k	25.79 (10.97)	27.50 (14.00)		7.11 (6.64)	5.50 (9.00)		40.13 (5.10)	41.00 (5.00)		21.11 (5.96)	21.00 (8.00)	
	$80k–$100k	25.89 (11.86)	22.00 (22.00)		4.04 (3.27)	3.00 (3.00)		39.54 (5.89)	40.00 (10.00)		21.22 (7.22)	21.00 (10.00)	
	>$100k	22.50 (12.59)	21.00 (18.00)		5.61 (5.70)	4.00 (5.00)		40.00 (5.76)	41.00 (8.00)		20.07 (6.94)	19.50 (10.00)	
Gross household annual income	<$100k	26.59 (10.11)	29.00 (15.00)	0.83^b^	6.30 (5.17)	5.00 (7.00)	0.18^b^	39.93 (4.48)	40.00 (6.00)	0.88^b^	20.93 (7.89)	21.00 (10.00)	0.68^b^
	$100k–$150k	25.21 (13.89)	23.50 (22.00)		7.41 (6.68)	5.50 (8.00)		38.09 (7.30)	40.50 (8.00)		22.03 (7.11)	22.00 (9.00)	
	$150k–$200k	24.61 (12.26)	21.00 (21.00)		5.04 (5.51)	3.00 (6.00)		40.36 (4.62)	40.50 (8.00)		21.32 (6.60)	21.00 (10.00)	
	>$200k	24.87 (11.14)	22.00 (12.00)		5.13 (5.79)	3.00 (6.00)		39.61 (6.04)	40.00 (7.00)		19.35 (5.53)	20.00 (8.00)	

^a^ *p*‐value from parametric test.

^b^ *p*‐value from non‐parametric test.

**TABLE 4:** Multiple regression analyses predicting likelihood of burnout domains and occupational stress prevalence.

Variable		B (95% CI)	Beta	r	*p*
Emotional Exhaustion					
Intercept		7.66 (−4.47, 19.78)			0.213
Physically active^e^		−5.33 (−10.95, 0.30)	−0.17	−0.16	0.063
Work sector^a^	Public^f^	8.50 (−3.60, 20.60)	0.18	0.12	0.166
	Private practice^g^	9.93 (0.38, 19.47)	0.35	0.17	**0.042**
	Combination^h^	14.89 (3.89, 25.89)	0.41	0.22	**0.008**
Work isolation^i^		5.62 (1.37, 9.88)	0.23	0.22	**0.010**
Work hours^b^	30–40 hours^j^	8.23 (1.72, 14.74)	0.34	0.21	**0.014**
	40–50 hours^k^	12.69 (6.17, 19.20)	0.53	0.32	**<0.001**
	>50 hours^l^	14.52 (6.33, 22.71)	0.39	0.29	**<0.001**
Depersonalisation					
Intercept		0.84 (−2.32, 3.99)			0.601
Caseload^m^		3.36 (1.22, 5.50)	0.28	0.27	**0.002**
Work hours^b^	30–40 hours^j^	4.62 (1.16, 8.07)	0.38	0.24	**0.009**
	40–50 hours^k^	3.62 (0.20, 7.04)	0.30	0.19	**0.038**
	>50 hours^l^	7.18 (2.83, 11.53)	0.38	0.29	**0.001**
Personal accomplishment					
Intercept		38.78 (35.88, 41.68)			<0.001
Ethnicity^c^	Māori^p^	−4.12 (−9.26, 0.42)	−0.17	−0.17	0.073
	Asian^q^	−3.67 (−7.13, −0.21)	−0.20	−0.19	**0.038**
	Other^r^	−3.01 (−6.15, 0.13)	−0.18	−0.18	0.060
Physically active^e^		2.77 (−0.12, 5.66)	0.18	−0.18	0.060
Caseload^m^		−1.65 (−3.88, 0.59)	−0.14	−0.13	0.147
Occupational stress					
Intercept		19.56 (13.96, 25.16)			<0.001
Physically active^e^		−2.01 (−7.70, 3.67)	−0.10	−0.10	0.479
Work sector^d^	Public^f^	8.09 (1.82, 14.36)	0.36	0.35	**0.013**
	Research/education^n^	3.14 (−3.55, 9.83)	0.12	0.13	0.349
	Combination^h^	2.40 (−3.26, 8.05)	0.13	0.11	0.397
Management responsibility^o^		5.58 (1.21, 9.96)	0.37	0.34	**0.014**

*Note*: Bolded values are reflective of significant probability values.

Abbreviations: CI = confidence interval.

^a^ Compared to research/education reference category.

^b^ Compared to <30 h reference category.

^c^ Compared to NZ European reference category.

^d^ Compared to private sector reference category.

^e^ Coded 1 = physically active, 0 = not physically active.

^f^ Coded 1 = works in public sector, 0 = works in other sectors.

^g^ Coded 1 = works in private practice, 0 = works in other sectors.

^h^ Coded 1 = works in a combination of sectors, 0 = works in other sectors.

^i^ Coded 1 = works alone, 0 = works with other practitioners.

^j^ Coded 1 = works between 30 and 40 h per week, 0 = works other hours.

^k^ Coded 1 = works between 40 and 50 h per week, 0 = works other hours.

^l^ Coded 1 = works more than 50 h per week, 0 = works other hours.

^m^ Coded 1 = works in all specialities (dermatology, musculoskeletal, diabetic, rheumatology); 0 = works in one or two specialities.

^n^ Coded 1 = works in research/education, 0 = works in other sectors.

^o^ Coded 1 = no management responsibility, 0 = management responsibility.

^p^ Coded 1 = identifies as Māori, 0 = identifies as alternative ethnicity.

^q^ Coded 1 = identifies as Asian, 0 = identifies as alternative ethnicity.

^r^ Coded 1 = identifies as other, 0 = identifies as alternative ethnicity.

The following variables were entered into the final regression model for emotional exhaustion: physical activity status, work sector, work isolation and work hours, (*R* ^*2*^ = 0.304), *F* (8, *N* = 110) = 5.519, *p* < 0.001. Working in private practice, a combination of work sectors, work isolation and all weekly work hour categories remained significant when entered into the model. Being physically active and working in the public sector did not significantly contribute to the final model. The estimates indicate that practitioners working in the private sector or in a combination of sectors are more emotionally exhausted compared to those who work in the research/education sector (reference category). Practitioners who worked in isolation also had higher emotional exhaustion compared to those who worked with other practitioners. Finally, practitioners who worked more than 30 h per week had higher emotional exhaustion compared to those who worked less than 30 h.

Caseload and weekly work hours were entered into the final regression model for depersonalisation, (*R* ^*2*^ = 0.183), *F* (4, *N* = 108) = 5.770, *p* < 0.001. Both variables significantly contributed to the model. The estimates indicate practitioners with a more varied caseload had higher levels of depersonalisation compared to those who worked in only one or two areas of speciality. Practitioners who worked more than 30 h a week had higher levels of depersonalisation compared to those who worked less than 30 h per week.

Ethnicity, physical activity status and patient caseload were entered into the final regression model for personal accomplishment, (*R* ^*2*^ = 0.152), F (5, *N* = 106) = 3.577, *p* < 0.005. Ethnicity was the only significant contributor to the model. Practitioners that identified as Asian had significantly lower levels of personal accomplishment compared to NZ European practitioners (reference category). Although not statistically significant, practitioners who participated in physical activity had higher levels of personal accomplishment and those who worked across a variety of specialities were prone to lower levels of personal accomplishment compared to practitioners who worked across one or two specialities.

Physical activity, work sector and management responsibility were entered into the final regression model for occupational stress, (*R* ^*2*^ = 0.282), *F* (5, *N* = 47) = 3.218, *p* = 0.15. Of the variables entered within the model, working in the public sector and having management responsibility made a statistically significant contribution. Practitioners who were physically active had lower stress compared to those who were not physically active. Practitioners working in the public sector demonstrated higher levels of stress compared to those working in private practice. Research/education and combination sectors also demonstrated higher levels of stress compared to private practice. Practitioners with managerial responsibility had higher levels of stress compared to those in non‐management roles.

### 3.4 Aim 3: Examine the relationship between burnout risk and occupational stress

The relationship between stress and each of the three burnout domains was investigated using the non‐parametric Spearman's rank‐order correlation coefficients. There was a strong positive correlation between stress and emotional exhaustion, *rho* = 0.59, *N* = 111, *p* < 0.001, with high levels of emotional exhaustion associated with high levels of stress. A medium negative correlation was found between personal accomplishment, with low levels of personal accomplishment associated with high levels of stress, *rho* = −0.33, *N* = 111, *p* < 0.001. Finally, there was a small positive correlation between depersonalisation and stress, *rho* = 0.22, *N* = 111, *p* = 0.02.

## 4 DISCUSSION

This study was the first to explore burnout risk and occupational stress among podiatrists in AoNZ. The findings from this study demonstrate a notable number of podiatrists exhibiting moderate to high risk of developing burnout and some experiencing severe to dangerous levels of occupational stress. Moreover, various modifiable and non‐modifiable factors related to demographic and professional characteristics were associated with the development of burnout and stress.

Over half of respondents demonstrated stress at moderate, severe or dangerous levels, with a quarter of participants experiencing moderate to high levels of overall burnout risk. This finding is consistent with a recent study in which 35% of Australian podiatrists exhibited moderate to severe burnout risk and, due to trans‐Tasman practice agreements, may be considered closely aligned with practice in AoNZ [11, 28]. Overall, the current study has shown that podiatrists in AoNZ experience high levels of emotional exhaustion. The higher prevalence of this burnout domain is also observed within UK and Australian podiatric burnout studies [10, 11]. Conceptually, emotional exhaustion is believed to be the first domain to develop in response to stress and has been considered the core component of burnout [29, 30]. This notion supports the later development of depersonalisation and reduced personal accomplishment which may explain the lower prevalence of these domains observed among practitioners.

Various demographic factors were associated with each burnout domain and occupational stress. Firstly, working in isolation significantly predicts high levels of emotional exhaustion and is a known primary outcome of workplace social isolation [31]. As almost two‐fifths of practitioners work entirely independently, this poses a considerable risk to burnout development among this group and may account for the overall prevalence of high emotional exhaustion.

Working in private practice or across a combination of work sectors also predicted higher levels of emotional exhaustion among practitioners. However, working in the public sector indicated higher stress levels than working in private practice. The inverse result would be expected when considering the proposed development from occupational stress to burnout (i.e., higher stress levels in the private sector compared to the public sector). This result may be explained by the difference in stressor exposures between practitioners working within each sector such as caseload complexity, professional isolation and work hours. However, more research is needed to explore the impact of work settings within the podiatry profession.

Practitioners who worked more than 30 h per week exhibited higher levels of emotional exhaustion and depersonalisation than those who worked less than 30 h. This statistic is expected, as studies corroborate that increased working hours are significantly related to burnout [32]. The high number of practitioners working more than full‐time hours (>40 h per week) may result from more than half of respondents owning their own business. Additionally, the workforce shortages currently faced by the podiatry profession may further exacerbate workload issues for business owners, with difficulties in the recruitment of podiatrists in an attempt to mitigate their high workloads [9].

Patient caseload was a significant contributor to high depersonalisation. The podiatry profession can be considered a specialised, yet highly varied profession in terms of clinical outputs and capabilities. Podiatrists are required to exhibit a wide range of expertise and clinical skill sets to effectively treat and manage a myriad of lower limb presentations. Clinicians with a varied caseload demonstrated higher levels of depersonalisation compared to those who only work in one or two areas of speciality. This may be attributed to the broader and more varied knowledge and skill sets required to treat all patient presentations effectively and efficiently. Practitioners working across all specialities may be exposed to areas of podiatry they do not enjoy or feel confident in, subsequently reducing esteem, requiring greater focus and developing feelings of detachment and depersonalisation to cope [33].

The high correlation observed in the current study between emotional exhaustion and stress aligns with the Maslach's theoretical model of burnout development, meaning emotional exhaustion is the first domain to develop in response to chronic stress [2]. Further supporting this relationship, Stordeur (2001) performed multivariate analyses between work stressors and emotional exhaustion on hospital nursing staff, demonstrating increased variance compared to other variables [34]. A 2012 study exploring stress and burnout within emergency departments also confirmed the association between various sources of stress with emotional exhaustion and depersonalisation; however, it was less significant with reduced personal accomplishment [35]. McManus et al. (2002) found that depersonalisation reduced stress, reportedly through an ego‐defence mechanism [36]. In contrast, personal accomplishment increased stress levels and indirectly increased emotional exhaustion [36]. Due to the cross‐sectional nature of this study, it is important to consider that the causation between the two variables was unable to be determined (i.e., if stress causes emotional exhaustion or if emotional exhaustion causes stress).

The following limitations should be considered when interpreting the findings from this study. The successful recruitment of participants captured data from 24% of those holding a current annual practising certificate; however, those experiencing the harmful emotional, physical and mental effects of burnout may be more or less inclined to participate due to the voluntary nature of the survey. As this was a self‐reported survey, systemic and non‐response biases can also influence data and interpretation [37]. Therefore, this data may not be generalisable to all AoNZ podiatrists. Secondly, younger practitioners (ages 20–30) and those with less than five years of practice experience were underrepresented among respondents. Previous survey data by the Association of NZ Podiatrists and burnout studies on new graduate podiatrists support the notion that burnout levels among this cohort may be higher than reflected in this study [10, 38]. Finally, qualitative data were not collected in this study. Future qualitative research exploring the perceptions and experiences of burnout among podiatrists may further enhance our understanding of the challenges facing this profession.

## 5 CONCLUSION

This is the first formal investigation into burnout and occupational stress among the AoNZ podiatry workforce. The results from this study have shown that burnout risk and occupational stress are prevalent among AoNZ podiatrists and are associated with a number of modifiable factors, including high work hours and collegial isolation. Furthermore, emotional exhaustion was found to be strongly correlated with stress. Further research is required to improve understanding of this issue to implement prevention strategies and maintain optimal foot care services within AoNZ.

## AUTHOR CONTRIBUTIONS

**Mia Clarke:** Conceptualization and design; data analysis; interpretation of data; writing – original draft. **Mike Frecklington:** Conceptualization and design, interpretation of data, writing – review & editing. **Sarah Stewart:** Conceptualization and design, data analysis, interpretation of data, writing – review & editing.

## CONFLICT OF INTEREST STATEMENT

None of the authors have any conflicts of interest to declare.

## ETHICS STATEMENT

This study was approved by the Auckland University of Technology Ethics Committee.

## Supporting information

Supporting Information S1

## Data Availability

The data that support the findings of this study are available in the supplementary material of this article.
