# Supplementary material for: Burnout among the podiatry profession: A survey of podiatrists in Aotearoa New Zealand
Source: J Foot Ankle Res. 2024 Jun 10;17(2):e12030. doi: 10.1002/jfa2.12030 (PMC11296713; doi:10.1002/jfa2.12030)
Supplement: Supplementary file 1 — Supporting Information S1 [file JFA2-17-e12030-s001.docx]

**Supporting information 1:** Survey

**Personal and professional demographic information**

What gender do you identify as?

Male

Female

Gender diverse

Prefer to self-describe:

What is your age?

20-25 years

26-30 years

31-35 years

36-40 years

41-45 years

46-50 years

51-55 years

56-60 years

61-65 years

66 years or older

Which race or ethnicity best describes you? (Please choose only one)

NZ/European

Māori

Asian

Pasifika

Middle Eastern

Latin American

African

Other

What is your marital status?

Single (never married)

Married or in a de facto partnership

Divorced/seperated

Widowed

Prefer not to say

How many children or dependants do you have?

None

1

2-4

More than 4

Prefer not to say

On average, how many hours sleep do you get a night?

Less than 4 hours

4-6 hours

6-8 hours

8 hours or more

Do you affiliate with religion?

Yes

No

Prefer not to say

What religion do you affiliate with? (optional)

Do you set aside time to undertake physical activity/exercise each week?

Yes

No

I'm not sure

On average how many hours per week would you be physically active?

Less than 1 hour

1-2 hours

3-4 hours

More than 4 hours

Do you practice mindfulness such as meditation, breathing exercises or yoga?

Yes

No

I'm not sure

On average, how many hours would you practice this for?

How many years have you practiced as a podiatrist?

Less than 2 years

2-5 years

5-10 years

10-15 years

15-20 years

20 years or more

What is the highest level of formal education you have completed?

Bachelor's degree

Postgraduate certificate/diploma/bachelor’s honours degree

Master's degree

Doctoral degree

Are you currently enrolled in postgraduate study?

Yes

No

Which of the following statements best describes your work as a podiatrist? (You can select more than one answer)

Private practice

Public

Research

Education

☐ Other (please specify)

Do you own or co-own your place of employment?

☐ Yes

☐ No

Do you have any other management roles or responsibilities?

☐ Yes

☐ No

Which of the following statements best describes your daily workplace?

I primarily work by myself most of the time

I primarily work with other podiatrists most of the time

I primarily work with other health professionals most of the time

I primarily work with other health professionals and other podiatrists most of the time

What environment to you primarily work in?

Rest homes

Mobile podiatry/home visits

Private clinic

Hospital or out service clinics

University or Research Centre

Other (please specify)

What best describes your patient case load?

Mostly dermatological care

Mostly musculoskeletal care

Mostly diabetic and rheumatological care

A combination of all three

A combination of diabetic/rheumatological care and dermatological care

A combination of dermatological care and musculoskeletal care

A combination of musculoskeletal care and diabetic/rheumatological care

I work in a university or research centre and do not have a patient case load

On average, how many hours do you work a week?

Not currently working (maternity leave, LWOP, between jobs, sabbatical etc)

Less than 10

10-20 hours

20-30 hours

30-35 hours

35-40 hours

40-45 hours

45-50 hours

50 hours or more

On average, how many hours of patient contact do you have per day?

0-1 hours

1-3 hours

4-7 hours

8 hours or more

What region/location do you primarily practice in?

Northland

Auckland

Waikato

Bay of Plenty

Gisborne

Hawkes Bay

Taranaki

Manawatu-Wanganui

Wellington

Marlborough

Nelson

Tasman

West Coast

Canterbury

Otago

Southland

Chatham Islands

What is the average time spent commuting per day from your home residence to your primary workplace?

10 or less minutes

10-20 minutes

20-30 minutes

30-45 minutes

45-60 minutes

60-90 minutes

Over 90 minutes

What is your gross annual income?

$60,000 or less

$60,000 - $80,000

$80,000 - $100,000

$100,000 or more

What is your gross annual household income?

My income is the only one generated for myself or my family

$100,000 or less

$100,000 - $150,000

$150,000 - $200,000

$200,000 - $250,000

$250,000 - $300,000

$300,000 or more

**Maslach Burnout Inventory: Section A**
For each of the following statements please indicate how often this applies to you.

Never (0); A few times per year (1); Once a month (2); A few times per month (3); Once a week (4); A few times per week (5); Every day (6)

I feel emotionally drained by my work.

Working with people all day long requires a great deal of effort.

I feel like my work is breaking me down.

I feel frustrated by my work.

I feel I work too hard at my job.

It stresses me too much to work in direct contact with people.

I feel like I'm at the end of my rope.

**Maslach Burnout Inventory: Section B**

For each of the following statements please indicate how often this applies to you.

Never (0); A few times per year (1); Once a month (2); A few times per month (3); Once a week (4); A few times per week (5); Every day (6)

I feel I look after certain patients/clients impersonally, as if they are objects.

I feel tired when I get up in the morning and have to face another day at work.

I have the impression that my patients/clients make me responsible for some of their problems.

I am at the end of my patience at the end of my day at work.

I really don't care about what happens to some of my patients/clients.

I have become more insensitive to people since I've been working.

I'm afraid that this job is making me uncaring.

**Maslach Burnout Inventory: Section C**

For each of the following statements please indicate how often this applies to you.

Never (0); A few times per year (1); Once a month (2); A few times per month (3); Once a week (4); A few times per week (5); Every day (6)

I accomplish many worthwhile things in this job.

I feel full of energy.

I am easily able to understand what my patients/clients feel.

I look after my patients'/clients' problems very effectively.

In my work, I handle emotional problems very calmly.

Through my work, I feel that I have a positive influence on people.

I am easily able to create a relaxed atmosphere with my patients/clients.

I feel refreshed when I have been close to my patients/clients at work.

**The Workplace Stress Scale**

Thinking about your current job, how often does each of the following statements describe how you feel?

Never (0); A few times per year (1); Once a month (2); A few times per month (3); Once a week (4); A few times per week (5); Every day (6)

Conditions at work are unpleasant or sometimes even unsafe.

I feel that my job is negatively affecting my physical or emotional well-being.

I have too much work to do and/or too many unreasonable deadlines.

I find it difficult to express my opinions or feelings about my job conditions to my superiors.

I feel that job pressures interfere with my family or personal life.

I feel that I have inadequate control or input over my work duties.

I receive inadequate recognition or rewards for good performance,

I am unable to fully utilize my skills and talents at work.
